# Insights into maternal pertussis vaccination counselling: a qualitative study on perspectives and experiences among midwives and gynaecologists in the Netherlands

**DOI:** 10.1186/s12879-024-09681-7

**Published:** 2024-09-02

**Authors:** Veja Widdershoven, Eveline C.H. van Eerd, Marije Pfeyffer, Liesse M.L. Vanderhoven, Amanja Verhaegh-Haasnoot, Rianne P. Reijs, Christian J.P.A. Hoebe

**Affiliations:** 1 Department of Sexual Health, Infectious Diseases and Environmental Health, Living Lab Public Health Mosa, South Limburg Public Health Service, PO Box 33, Heerlen, 6400 AA The Netherlands; 2 Department of Social Medicine, Care and Public Health Research Institute (CAPHRI), Maastricht University, PO Box 616, Maastricht, 6200 MD The Netherlands; 3 Department of Youth Health Care, Living Lab Public Health Mosa, Public Health Service South Limburg, Heerlen, The Netherlands; 4 Department of Microbiology, Infectious Diseases and Infection Prevention, Care and Public Health Research Institute (CAPHRI), Maastricht University Medical Centre (MUMC+), PO Box 5800, Maastricht, 6202 AZ The Netherlands

**Keywords:** Maternal pertussis vaccination, Counselling, Healthcare professionals, Qualitative study

## Abstract

**Background:**

Healthcare professionals (HCPs) play a significant role in the decision-making process of pregnant women on maternal vaccinations. Whereas a high proportion of HCPs discuss maternal vaccinations with pregnant women, confidence in discussing maternal vaccinations is lacking and HCPs experience inadequate training to discuss maternal vaccinations with pregnant women. Furthermore, different practical barriers might influence the consultation process, such as lack of time. More studies on the barriers, as well as facilitators, to discussing maternal vaccinations is needed and will help us to better understand and support HCPs in discussing maternal vaccinations.

**Methods:**

This qualitative study involved semi-structured interviews with fourteen HCPs working as midwives or gynaecologists in the Netherlands. An integrated theoretical approach was used to inform data collection and analysis. Thematic analysis was conducted using inductive and deductive approaches. This study followed the COnsolidated criteria for REporting Qualitative research (COREQ) guidelines.

**Results:**

The thematic analysis of the data pointed to the following five themes of HCP counselling: the consultation process, attitude, perceived norm, perceived control and improvement ideas. Most HCPs follow a similar approach in maternal pertussis vaccination consultations, beginning by assessing clients’ understanding, providing basic information, and addressing questions. However, consultation timing and prioritization vary among HCPs. Challenges in consultations include client requests for clear advice, with HCPs trained to remain neutral, emphasizing client autonomy in decision-making. Most HCPs acknowledge the importance of their consultations in informing pregnant women about maternal pertussis vaccination.

**Conclusions:**

This study offers a confirmation of the awareness of the pivotal role of HCPs in informing pregnant women about the maternal pertussis vaccination. HCPs stress the importance of neutral counselling, enabling pregnant women to make well-informed decisions independently. Because of upcoming vaccine hesitancy nowadays, HCPs must be equipped with the knowledge and confidence to navigate difficult conversations. Continuous education and training might help to increase HCPs’ confidence in handling difficult consultations. Additionally, making the information materials for pregnant women available in multiple languages and incorporating more visuals to enhance comprehension could support HCPs in reaching a broader group of pregnant women.

## Introduction

Maternal immunization is an effective strategy in preventing pregnant women and their newborns from severe diseases, like influenza and pertussis [1–3]. Previous studies have demonstrated the significant impact of maternal immunization, resulting in a 63% reduction in influenza cases and a 91% decrease in pertussis infections among young infants [4, 5]. The Dutch National Immunization Program (NIP) introduced the maternal pertussis vaccination (MPV) in December 2019. This followed earlier implementations in the UK (2012), Australia (2015), Belgium (2013) and US (2012) [6–10]. In the Netherlands MPV is given by the Public Health Service (PHS) as part of Youth Health Care. Midwives and gynaecologists are expected to briefly discuss the possibility of MPV, handing out the national information leaflet and referring pregnant women to the website for more information and for making an appointment between 14 and 22 weeks of pregnancy. These healthcare professionals (HCPs) are not involved in actual vaccine delivery. Notwithstanding the recognized public and individual health advantages of MPV, the coverage among pregnant women is lower than anticipated; about 61% in the UK, 55% in the US and 64% in the Netherlands in 2022 [11–13]. Many pregnant women remain hesitant towards receiving the MPV, making the task of increasing acceptance a global challenge [14–16].

Studies investigating reasons related to MPV acceptance and refusal have shown that HCPs, mainly midwives and gynaecologists, play a significant role in the decision-making process of pregnant women [3, 16–18]. Receiving a HCP recommendation is a main facilitator of increasing MPV acceptance, while absence of this recommendation increases vaccine hesitancy and is one of the main barriers reported among unvaccinated pregnant women [15, 17–21]. In addition, inadequate knowledge is also a barrier to vaccination acceptance. Such as knowledge about the vaccine, for example their efficacy and availability, and knowledge about the diseases they prevent [17, 18]. For many pregnant women this knowledge is only gained when the vaccination is discussed with a HCP [17]. In our previous questionnaire study among MPV acceptors and refusers in the Netherlands, 13% of MPV acceptors indicated that they did not know MPV existed until their midwife or obstetrician provided information about the vaccination. Moreover, 52% of MPV acceptors mentioned that they accepted MPV because of the information given by a HCP, underlying the importance of HCPs as a source of information for pregnant women [22]. During pregnancy, women have frequent contact with their midwife or gynaecologist and therefore information and recommendations about MPV can easily be given.

Although a high proportion of HCPs discuss maternal vaccinations with pregnant women, studies have suggested that they lack confidence in these discussions and often experience inadequate training for discussing maternal vaccinations with pregnant women [16, 18, 21, 23]. In addition to insufficient training, lack of knowledge about the disease and the vaccines themselves is a significant barrier to discussing maternal vaccinations [23, 24]. Several studies have identified that HCPs can possess negative attitudes towards maternal vaccinations, often due to doubts about the effectiveness and safety of administering vaccines during pregnancy. These doubts were found to be more prevalent among midwives compared to other HCPs [17, 21, 25]. Furthermore, various practical barriers have been cited. HCPs frequently experience uncertainties regarding who is responsible for informing pregnant women about maternal vaccinations. Additionally, a high workload and consequent lack of time significantly impact their ability to discuss maternal vaccinations adequately [18, 21, 23, 26]. Concerns about reimbursement for the time spent discussing maternal vaccinations further complicates these interactions [18]. More research on both the barriers and facilitators of discussing maternal vaccinations is needed to better understand and support HCPs in discussing maternal vaccinations.

The Reasoned Action Approach (RAA) is used in this study to understand the behaviour of HCPs with regards to discussing maternal vaccinations. According to the RAA, behaviour is best predicted by the intention to perform that behaviour. The theory identifies three main determinants for explaining and predicting intention. First, the attitude a person has towards a particular behaviour. Second, the normative influence, which represents the perceived social pressure towards a particular behaviour. Third, a person’s perceived behavioural control, which reflects the belief in their skills and abilities to execute the behaviour. In addition to these determinants, the RAA emphasizes the significant influence of skills and environmental barriers a person may encounter in executing a particular behaviour [27]. This qualitative study explores the perspectives and experiences of midwives and gynaecologists in the Netherlands regarding MPV counselling through semi-structured interviews. The aim is to uncover barriers and facilitators that can eventually inform interventions to optimize maternal vaccination advice by HCPs and to increase maternal vaccination uptake.

## Methods

### Study design

To explore the perspectives of HCPs concerning MPV counselling, a theory-informed qualitative study was conducted involving semi-structured interviews. We assessed the perspectives of HCPs on MPV counselling partially by assessing determinants of the Reasoned Action Approach (RAA). The RAA aims to explain and predict behaviour, in our study MPV counselling, based on attitudes, perceived norms and perceived behavioural control [28]. Additionally, the RAA considers required skills or environmental barriers that influence whether someone’s intention is converted into behaviour. The COnsolidated criteria for REporting Qualitative research (COREQ) guidelines were followed for data reporting [29].

### Participant selection

Participants were HCPs working as midwives or gynaecologists in the Netherlands. Some participants were recruited via a previous online cross-sectional questionnaire study [30] about MPV counselling. HCPs who indicated having interest in participating in an interview, received an invitation for the interview via e-mail. Invited participants were asked to recruit future participants among their co-workers, using the snowball principle. When HCPs were willing to participate, the interview was scheduled and an informed consent form was signed. Participants were recruited until data saturation was achieved.

### Data collection

Semi-structured audio-recorded interviews with HCPs were conducted between February 2022 and February 2023. The interviews lasted approximately 30–40 min each and were conducted online or by telephone. Interviews were held by experienced interviewers: a female youth health care physician (MP) and a female PhD student (VW). Prior to the study, no relationship was established between the participants and the interviewers. At the beginning of the interview, the interviewers introduced themselves and the aim and purpose of this study were explained once more. Confidentiality of data was emphasised to minimize possible socially desirable answers.

Interviews were audio recorded, transcribed verbatim and anonymized. An interview guide consisting of several main themes was developed a priori. MP and VW developed this guide based on a previous questionnaire study among HCPs about MPV counselling using the theoretical framework developed by Visser et al. [31]. Questions asked in the current qualitative study were used to gain more in depth perspectives on MPV counselling and related perceived barriers and facilitators. Themes included were: consultation processes and practical implications (when and how MPV counselling takes place), attitudes towards MPV and MPV counselling, barriers and facilitators to MPV counselling, and improvement ideas. No pilot interview was conducted.

### Data analysis

**Fig. 1:**
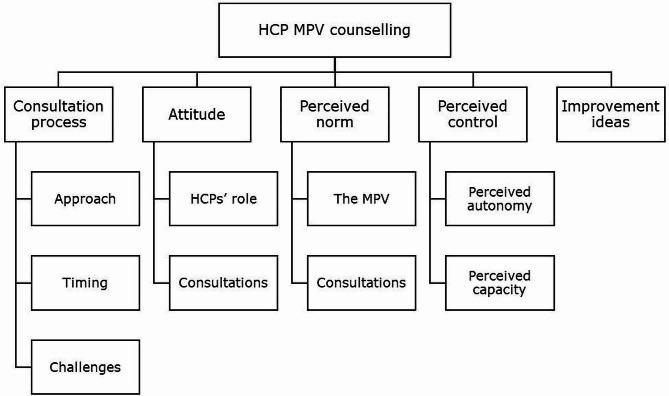
Coding tree based on the Reasoned Action Approach

Interviews were transcribed verbatim in Dutch by an external transcription service company. The transcripts were imported into ATLAS.ti 23 software for qualitative analysis. A thematic analysis was performed, using the 15-point checklist of criteria for good thematic analysis developed by Braun and Clarke [28]. We used a hybrid process of inductive and deductive coding. An initial coding structure was developed by two researchers based on the RAA and themes from the interview guide (Fig. 1). In addition to the initial coding structure, emergent codes were added by using inductive coding. The coding process was conducted iteratively, persisting until no additional codes emerged. In addition, field notes were compared with transcripts to develop a deeper understanding of the interpretation of the data. The field notes helped to collect contextual data and identify meaningful, expressive phrases, body language, and emotions in interview passages during the process of coding. Thereby, our analysis focussed on both explicit and implicit dimensions of the qualitative data, providing a more nuanced and comprehensive analysis. Data analysis was performed by two independent researchers (EE and VW). Discrepancies during the coding process were discussed among the researchers until a consensus was reached.

### Ethics statement

This study was approved by the Medical Ethical Committee of the University of Maastricht (METC 2020-2296-A-1).

## Results

In total, 79 HCPs were personally approached via e-mail or telephone. Of the invited HCPs, 14 one-time interviews (18%) were conducted over the course of the twelve month research period. The participants consisted of 10 midwifes and 4 gynaecologists from 5 different provinces in the Netherlands. The majority of them were female (93%, *n* = 13).

Results will be described based on the coding tree (Fig. 1), starting with the overall consultation process, followed by attitude, perceived norm, perceived control and improvement ideas.

### Overall consultation process

#### Consultation approach

The majority of HCPs expressed a similar approach to their consultation sessions. They often started by asking pregnant women, hereafter usually referred to as clients, about their prior understanding of MPV, followed by providing basic information and answering questions: *“So I ask […] have you ever heard of the maternal pertussis vaccination? Do you know what it means? We also give them the flyer […] from the National Institute for Public Health and the Environment (RIVM). […] And then [I] write down a number they can call for [making the] appointment.” (P4*,* midwife).* Most HCPs used two common arguments during their consultations. First, they explain that the baby is protected against the harmful effects of a pertussis infection starting at birth as the baby receives antibodies from the mother. Second, this means that, in most cases, the newborn needs one vaccination less. A few HCPs provided limited information about MPV because they feel it is not their responsibility and they receive no extra time or money for discussing it: *“I explain very little about this [MPV] […] because there is a discussion about who is responsible [for providing information].” (P11*,* midwife).*

At the end of the consultation, HCPs would hand out the RIVM flyer and explain how to make the appointment online. Notably, all HCPs indicated not distributing flyers in languages other than Dutch or English. In circumstances where there was a language barrier, HCPs referred their clients to the official RIVM website: *“If I really have to*,* I refer to the RIVM [website] where the translation can be found*,* but I have not printed [the flyers] out myself […].” (P2*,* midwife)*. Two HCPs stated that they just referred clients to the website and did not hand out flyers, citing environmental concerns in one case.

#### Consultation timing

The vaccination consultation takes about 5 to 10 min on average, depending on the client’s prior knowledge and their questions or concerns. The timing of the consultation sessions differed substantially across the HCPs. The most often agreed-upon schedule was to do an initial consultation session and hand out the flyer in week 16, followed by a follow-up session in week 22 to address further questions and enquire about a possible vaccination appointment: *“At 16 weeks we give the information about [the vaccine]*,* and then […] we’ll come back to that after those 22 weeks […].” (P8*,* midwife). “And at 22 weeks we verify whether they have had enough information*,* whether it was clear and whether they want to know anything else about [the vaccine]. And we note in the [medical file] whether they have made an appointment.” (P12*,* midwife).* Some HCPs, however, reported initiating the conversation as late as week 22 or 24, while others already mentioned the vaccine during intake in week 12 and revisited the matter in week 20. Few HCPs only discussed MPV during one consultation, unless the client addresses MPV herself again in a next consultation.

#### Challenges during consultations

HCPs mentioned some challenges that they face during their consultations. For example, some HCPs mentioned experiencing a challenge when clients request a clear positive or negative advice whether they should accept MPV. However, most HCPs stated that they had been trained to remain neutral while counselling: *“But they ask that [my opinion about MPV] very often. […] I always say*,* what I think*,* you do not think. […] And then I try to be like*,* but what do you think*,* why do you hesitate?” (P14*,* gynaecologist)*. Especially when clients were hesitant about MPV, many HCPs mentioned that they feel the importance to emphasize that the clients have their own choice to accept or reject MPV. HCPs would provide extra information if desired, but they never expressed their own preferences or choices: *“We counsel neutrally. If people really have questions about the vaccination*,* you answer these questions. You hope that you have given all the information and that all the questions have been answered. Then it [the choice] is up to the parent*,* I think. […] We are not going to push them or say you must make a choice” (P4*,* midwife).* Next to the neutral counselling, two HCPs acknowledged the importance of providing additional information or using drawings when counselling clients with a lower level of educational attainment. One HCP mentioned that occasionally MPV is not discussed during consultations due to different priorities, especially in case of complicated pregnancies.

### Attitude

#### Attitude regarding their role

Overall, most HCPs acknowledged the importance of their consultation and information in the decision-making process of pregnant women. Some HCPs addressed that they think pregnant women do not know about MPV if HCPs are not discussing it: *“I think it is important for pregnant women to know this [advantages MPV]*,* to be well informed.[…] You have to tell the pregnant woman [the existence of MPV]*,* because they do not know on their own.” (P1*,* gynaecologist).* They agree that only giving a flyer is not enough information and most of them mentioned the importance of repeating information about MPV: *“[…]the power of repetition is important*,* I think. […]For example like folic acid*,* that [communication] was really high for a while and everybody started taking folic acid even before they were pregnant. But that [awareness] is decreasing now.” (P7*,* midwife).*

Several participants have highlighted that the higher the quality of information and the more positive the HCP is towards the vaccine, the higher the vaccine uptake among pregnant women: *“what just appears from [previous research] is that the more positive the HCP is towards vaccinations*,* the greater the uptake. So*,* I just think this role [of doing the consultation] was made for us.” (P5*,* gynaecologist).* Nevertheless, the HCPs generally acknowledge the importance of pregnant women being free to make an informed choice about getting the MPV or not. One HCP reported that it is beneficial the obstetricians and gynaecologists are executing the consultations, as they are not working in an organization in favour of vaccinations, such as a Public Health Service (PHS): *“We [gynaecologists and obstetricians] play an important role*,* because they [pregnant women] do not see us as a PHS in favour of vaccinations. They see us as a caregiver for their baby.” (P14*,* gynaecologist).*

Overall, HCPs reported minimal opposition or criticism from clients during the MPV counselling. However, some HCPs mentioned that vaccinations are a sensitive topic nowadays, making it more difficult to discuss MPV. Clients often discovered information online before the consultation and formed their opinion about MPV: *“They [pregnant women] say that medication is not safe during pregnancy. But I happen to be the gynaecologist. I know that the vaccination is not bad. You should get this one [MPV].” (P9*,* gynaecologist).* Because HCPs want to keep a good relationship with the clients, they often gauge how the client thinks about MPV before providing any information. In addition, HCPs conversate more about maternal vaccinations such as COVID-19 and influenza vaccination.

#### Attitude regarding consultations

Timing and prioritizing of MPV consultations showed contrast between two groups of HCPs. Half of the HCPs expressed that counselling about MPV felt like a small effort that easily fits into their existing consultation time: *“I think it is easy to do at the 16-week consultation*,* we have fifteen minutes. […] So [counselling about MPV] fits in nicely*,* because it is one of the topics to discuss.” (P4*,* midwife).* The other half often prioritized other tasks over vaccination counselling due to the extensive nature of parental care: *„I already have so much in my consultation hours that I want to discuss” (P7*,* midwife).* Additionally, some HCPs felt dissatisfied about not being reimbursed for counselling MPV, while other HCPs considered counselling MPV to be minor in comparison to other responsibilities.

### Perceived norm

#### Perceived norm MPV

The majority of HCPs believed that most clients support and accept MPV. Even though HCPs know that some clients have a negative attitude towards MPV, have a fear of vaccinations, or have misconceptions about vaccinations (e.g. vaccination causes autism in children). Most HCPs reported that they do not encounter these clients during their consultations. However, some HCPs reported that MPV uptake might depend on the population (e.g. lower uptake in individuals with a lower level of educational attainment, and a lower socioeconomic status) and region. Several HCPs recognized that pregnant women based their decision on experiences and behaviour of family and friends (descriptive norm): *“During my consultations they say: My friend and sisters also received that [MPV]*,* thus so do I [accept MPV].” (P7*,* midwife)*. Besides, nowadays more and more information about MPV and maternal vaccinations are available online and in magazines. One HCP indicated that 80% of pregnant women simply follow the recommendation of the government without further consideration (injunctive norm): *“I think that 80% [of pregnant women] tend to accept MPV because it [MPV] is the recommendation of the government without further consideration. They just make an appointment.” (P3*,* midwife).*

#### Perceived norm consultations

While most HCPs have agreements within their medical office on when and how MPV will be discussed with the clients, there is uncertainty about whether these agreements are followed consistently across practitioners and healthcare settings (descriptive norm): *“We all hand out the flyer during the intake. [But] I do not really know to what extent we will come back to it at 20 weeks. I do know that we once agreed to come back to it one more time*,* but that was [long ago]*,* so to what extent do [the others] still do that?” (P3*,* midwife).* Several HCPs understood that other HCPs might not have the same opinion about the MPV and might therefore counsel differently due to different underlying beliefs. One participant said that she believed that there is a big group of HCPs that is not in favour of doing the MPV consultations and is therefore not doing them (well).

A key theme was the idea that MPV should be counselled as neutral as possible, highlighting the positive and negatives aspects of MPV without sharing their personal opinion. Most HCPs follow the injunctive principle that pregnant women should have autonomy and make their own decisions about maternal vaccinations. One HCP also emphasized feeling responsible for the delivery of information about MPV (injunctive norm): *“I feel like it is my responsibility to make sure they [pregnant women] are informed. […] If someone [pregnant woman] did not receive the MPV because she did not hear about it [MPV]*,* I would blame myself.” (P5*,* gynaecologist).*

### Perceived control

#### Perceived autonomy

A reoccurring topic was a lack of autonomy in establishing the terms and conditions of their engagement in MPV counselling. When MPV was newly introduced into the NIP, many HCPs felt that they were excluded from the discussions surrounding the implementation and only received information on the practicalities, leaving them with limited time to prepare. Midwives especially felt that this was yet another task that was imposed upon them without consulting them and without accompanying financial assistance: *“[…] when it [MPV] was first introduced*,* everyone was like*,* something gets added again that we have to counsel about*,* [but] which we are not allowed to do [vaccinate] ourselves. So again*,* a longer consultation time that you have to schedule.” (P8*,* midwife).* Some HCPs indicated that they would prefer to give MPV themselves or being compensated for counselling MPV to increase the sense of urgency and responsibility: *“And I do not really think [counselling the MPV] is my job […] I already have so much in my office hours that I want to discuss*,* […] But the moment you are also responsible for giving [the vaccine] yourself*,* I think that would make the counselling different.” (P7*,* midwife).*

Several HCPs expressed difficulties maintaining neutrality during MPV counselling, since the official advice from the government is to recommend the vaccine. HCPs were not allowed to express their own opinions about the decision to accept or reject MPV, which might limit them in choosing which information they provide.

#### Perceived capacity

Overall, HCPs feel competent in executing the consultation and answer frequently asked and basic questions. Yet, several HCPs expressed insecurities regarding answering difficult questions, e.g. about adjuvants in vaccines, or providing sufficient information when pregnant women are hesitant. Some HCPs found it easy to inform their clients that they would need to research the answer and will provide it at a later time: *“[…] most questions are doable. Otherwise*,* I say [that] I’m going to look it up [and] then I will come back to you. So*,* in that sense*,* always sure [about my ability to answer question]. And if I do not know*,* I’ll just look [the answer] up.” (P4*,* midwife).* Some HCPs mentioned sending clients to the PHS for more information or counselling, however, they feel that the PHS does not answer the questions either: *“[I] often notice that people ask questions*,* which […] I do not know the answer to either. [So] then I say*,* oh*,* just check with the PHS*,* because they are the ones giving the vaccination. But they [the PHS] often refer back to us because we should be doing the counselling.” (P3*,* midwife).* A few HCPs indicated that they do not know where to find more information and who to contact in case they have questions. Almost all HCPs expressed their wish for an extra training to refresh and update their knowledge about maternal vaccinations.

### Improvement ideas

All HCPs provided some improvement ideas that can be summarized in three categories: (1) information material for pregnant women, (2) increasing knowledge of HCPs and (3) administration of MPV. First, many HCPs mentioned the importance of having more inclusive information materials available for pregnant women. For example, providing information on various channels including forums, social media and local television channels watched by non-native speakers or having written information available in more languages and using visuals elements to make the information easier to understand: *“For example*,* for [information on] the NIPT (non-invasive prenatal testing) test [they use] very simple animations. Something like that would also be nice [for MPV]. Just also for the low literate*,* to whom visuals appeal more than linguistics.” (P5*,* gynaecologist).* Second, multiple HCPs mentioned introducing frequent training for HCPs to stay informed about MPV or other maternal vaccinations and to exchange practical examples: *“[…] sometimes I think it would be good for us to repeat [our knowledge of MPV] again [to refresh our memory].” (P7*,* midwife).*

Third, the majority of HCPs expressed their willingness to administer MPV themselves. The advantages they cited included reduced waiting time for appointments, elimination of the need for transportation to other locations, and greater ease in scheduling, particularly for individuals with low health literacy or low socio-economic status: *“I would like to give MPV myself. […] My assistant can make an appointment for you. There are no barriers*,* you do not have to make the appointment*,* you do not have to go somewhere else.” (P9*,* gynaecologist).* The disadvantages cited were: the large amount of administrative work and lacking the capacity to store the vaccination adequately in an official refrigerator with an expensive quality management system to secure the cold chain.

## Discussion

This study provides insights about HCPs’ perspectives and experiences concerning MPV counselling in the Netherlands. Our findings highlight that (1) HCPs are aware of their essential role in informing pregnant women about MPV, (2) HCPs stress the importance of neutral counselling, (3) HCPs express insecurities about providing accurate and comprehensive information, especially when faced with vaccine hesitant opinions, and (4) involvement of HCPs in the implementation process of MPV might increase the sense of urgency and responsibility.

Generally, midwives and gynaecologists have close relationships with their clients, establishing them as trusted sources of information [32, 33]. Pregnant women tend to be receptive to information provided by midwives and gynaecologists, making both the content (covering the benefits and risks of MPV) and the manner of delivery (through discussion or informational handouts) crucial factors [22, 34]. Multiple previous studies indicated a strong association between HCPs’ recommendation and MPV acceptance [33–35]. However, our study highlights that HCPs stress the importance of neutral counselling, enabling pregnant women to make well-informed decisions about MPV independently which contrasts with the clear positive advice by the government based on a high quality synthesis of evidence by the National Health Council. However, strong evidence implies that a recommendation by a HCP could increase maternal vaccination uptake [3, 15–21]. Therefore, it might be beneficial to shift the role of the HCP from a neutral counselling role to a positive advisory role. However, for this new role to be effective, it is important to consider the cultural environment in which the HCP operates and to ensure that a strong HCP-client relationship remains a priority.

HCPs have observed that vaccination has become a sensitive topic, especially after the COVID-19 pandemic, they express the increased difficulty to discuss MPV without damaging the relationship with their clients. Moreover, vaccine hesitancy is on the rise, prompting individuals to seek information online, where a large amount of confusing disinformation is present, consequently leading to an expanding body of misconceptions [35]. Thus, competent HCPs play a key role to answer difficult questions and provide accurate information that is needed to inform pregnant women. Several HCPs in our study expressed insecurities about providing accurate and comprehensive information, especially when faced with vaccine hesitant opinions. Regular training on topics such as vaccine ingredients, vaccine effectiveness and common misconceptions, might help to increase HCPs’ confidence and give them tools to handle difficult consultations about MPV. Additionally, providing information materials through several channels in multiple languages and incorporating more visuals to enhance comprehension could support HCPs in reaching a broader spectrum of pregnant women, including those who do not speak the native language and those with lower levels educational attainment.

While the majority of HCPs expressed a positive attitude about the MPV and their role in the decision-making process of pregnant women, some HCPs believed that it is not their responsibility to discuss MPV, partly due to the absence of a financial compensation or having insufficient time. This is bothersome as in the Dutch system the HCP referral role to the vaccination institute is essential as no national pregnancy register exists and therefore no central invitation system is available as is the case for other national vaccination programme vaccines. This lack hampers the ability to directly invite individuals for vaccination appointments. Establishing such a registry could improve vaccination uptake, as it appears that not all pregnant clients currently receive the necessary vaccination information. Furthermore, several HCPs expressed a desire for greater participation in the implementation process of MPV. Midwives in particular expressed that choices about the implementation were made without consulting them. This finding was confirmed by another Dutch qualitative study that aimed to evaluate the implementation of MPV [36]. Similar challenges have been observed in other European studies on the integration of maternal vaccinations into routine care for pregnant women. HCPs were found to have difficulties incorporating (the discussion of) maternal vaccinations into their standard care practices and lacked the confidence and knowledge to effectively inform their clients. Uncertainties about who is responsible for informing pregnant women about maternal vaccinations were observed as well. It was found that strong institutional support is necessary to help HCPs implement maternal vaccinations in their routine care, with a clear designation of responsibility [37, 38]. The absence of strong institutional support decreases the willingness, ability and sense of responsibility of HCPs to discuss MPV with pregnant women. Increasing engagement of stakeholders, such as HCPs, needs to be considered to decrease resistance to future guideline implementation [39]. More involvement in the implementation process of MPV might increase the sense of urgency and responsibility among HCPs and, thereby, increase the likelihood of HCPs prioritizing MPV counselling. Furthermore, a few HCPs provided limited information about MPV because they feel it is not their responsibility. As already mentioned in the introduction, the HCP is expected to briefly discuss the possibility of MPV, handing out the national information leaflet and referring pregnant women to the website for more information and for making an appointment between 14 and 22 weeks of pregnancy. It would be valuable to bring the guideline on the implementation of maternal vaccinations to the attention of gynaecologists and midwives in order to clarify their role and responsibilities in MPV counselling. In addition to involving HCPs in the decision-making processes surrounding MPV, compensating the professionals financially for their invested time could be beneficial for MPV uptake.

### Strengths and limitations

This study exhibits both strengths and limitations. One significant strength lies in the diverse study population, encompassing midwives and gynaecologists residing in 5 distinct Dutch provinces, with both female and male participants. A second strength is the coding process, carried out by two independent researchers. A third strength is that our study population included HCPs whose personal opinions were critical towards maternal vaccination. One expressed these concerns by not accepting the MPV during her own pregnancy. However, there are limitations to consider. One limitation pertains to the potential for selection bias, as some participants had previously responded to a questionnaire and expressed interest in participating in follow-up research. Despite this sampling process, data saturation was confirmed, as no new insights emerged. Additionally, the interviews were conducted either online or by telephone which could have potentially hindered effective communication.

## Conclusion

This study offers a confirmation of the awareness of the pivotal role of HCPs, particularly midwives and gynaecologists, in informing pregnant women about MPV who maintain close and trusted relationships with their clients. HCPs stress the importance of neutral counselling, enabling pregnant women to make well-informed decision independently. Shifting HCPs from neutral counselling to positive advising on MPV, could boost vaccination rates. However, discussions with pregnant women have become more complicated nowadays because of upcoming vaccine hesitancy. HCPs must be equipped with the knowledge and confidence to navigate difficult conversations, including addressing concerns about vaccine ingredients, effectiveness, and common misconceptions. HCPs express insecurities about providing accurate and comprehensive information. Regular education might help to increase HCPs’ confidence in handling difficult consultations. Additionally, information materials available in multiple languages and incorporating more visuals to enhance comprehension could support HCPs in reaching a broader group of pregnant women. Establishing a national registry for pregnant women, which can be used for direct MPV invitations could enhance vaccination rates. Furthermore, increasing engagement in the implementation process of future maternal vaccinations and providing financial compensation might increase HCPs sense of responsibility and the likelihood of prioritizing maternal vaccination counselling.

## Data Availability

The datasets generated and/or analysed during the current study are not publicly available due to the fact that the data of this study contain potentially identifying and sensitive participant information and that publicly sharing the data would not be in accordance with participant’s consent obtained for this study but are available from the corresponding author on reasonable request.

## Abbreviations

HCP: Healthcare professional
COREQ: Consolidated criteria for REporting Qualitative research
MPV: maternal pertussis vaccination
NIP: National immunization program
PHS: Public Health Service
RAA: Reasoned Action Approach
RIVM: National Institute for Public Health and the Environment
NIPT: non-invasive prenatal testing
